# Association between EN1 rs4144782 and susceptibility of knee osteoarthritis: A case-control study

**DOI:** 10.18632/oncotarget.16842

**Published:** 2017-04-05

**Authors:** Haohuan Li, Xiaolong Zhang, Yiping Cao, Song Hu, Fei Peng, Jianlin Zhou, Jianping Li

**Affiliations:** ^1^ Department of Orthopedics, Renmin Hospital of Wuhan University, Wuhan, 430060, China; ^2^ Key Laboratory of Optoelectronic Chemical Materials and Devices, Ministry of Education, Jianghan University, Wuhan, 430056, China; ^3^ Department of Physiology, Jianghan University, Wuhan Medical College, Wuhan, 430056, China

**Keywords:** osteoarthritis, EN1, polymorphism, BMD, case-control

## Abstract

Osteoarthritis (OA) is a complex disease that affects the whole joint, resulting from the combined influence of biomechanical factors and genetic factors. The heritable component for primary OA accounts for about 60% of variation in population liability to the disease. So far, genome-wide association studies (GWAS) and candidate gene studies have established many OA-related loci. However, these findings account for only a rather small fraction of the genetic component. To further reveal the genetic architecture of OA, we conducted this case-control study to explore the association of locus EN1 rs4144782 and knee OA susceptibility in a Chinese population. EN1 rs4144782 was significantly associated with increased risk of knee OA (OR=1.26; 95% CI: 1.05-1.50, P value=0.012). In dominant model, compared with carriers of GG genotype, those with AG or AA genotype have an 1.44-fold increased risk of OA (OR: 1.44; 95% CI: 1.10-1.88; P value=0.008). Subgroup analyses didn't change the results materially. This should be the first association study of EN1 locus on risk of OA, and our finding suggested that the EN1 rs4144782 might contribute to the susceptibility of knee OA.

## INTRODUCTION

The most common joint disorder, osteoarthritis (OA), which results from breakdown of joint cartilage and underlying bone, could cause substantial pain and physical disability [1]. However, no disease-modifying drugs exist for the treatment of OA [2]. To present, the concept of the pathophysiology is still evolving and undetermined, from being viewed as cartilage-limited to a multifactorial disease which could affect the whole joint [3]. Many genome-wide association studies (GWASs) of OA have been conducted in European Caucasian populations, and identified lot of susceptibility loci of OA consecutively, including LRCH1, PTGS2, A2BP1, 7q22, DOT1L, LSP1P3, and so on [4–14]. Candidate gene studies also identified the most consistently reported gene, GDF5, whose polymorphisms have been identified to be associated with susceptibility of OA [15–29]. These reports mean that genetic factors may play an important role in the development of OA, although just a little was identified.

Despite of numerous efforts to identify genetic factors associated with OA, the identified loci could still explain a small part of the hereditary of OA, due to the disease heterogeneity and limitation of large sample size [4, 13, 17, 27, 28, 30–36]. Recently, whole-genome sequencing identifies locus Homeobox protein engrailed-1 (EN1) gene as a determinant of bone mineral density (BMD) [37]. Higher BMD was reported to be associated with OA in many cross-sectional and longitudinal epidemiological studies [38–41]. One important mechanism that could underpin the observed association between BMD and OA is genetic pleiotropy—the existence of genetic variants that contribute to both BMD variation and OA risk [42]. For example, Yerges-Armstrong et al [43] evaluated the associations between GWAS-identified genetic loci for variance in BMD and knee OA, and identified four SNPs significantly associated with prevalent radiographic knee OA, including rs2016266, rs10226308, rs3736228, and rs10835187. This results further supported the hypothesis that BMD, or its determinants, might be a risk factor for OA development. Thus, to clarify the potential relationship between locus EN1 rs4144782 and knee OA susceptibility, we conducted this case-control study in a Chinese population.

## RESULTS

### Clinical characteristics between OA cases and healthy controls

Totally, we included 500 OA patients and 500 matched healthy controls in this study. As shown in Table 1, the proportion of the matching variable which included age, gender, smoking status, alcohol status, and regular physical activity in cases and healthy controls was comparable (all P value > 0.05). However, BMI in OA cases was larger than that in healthy controls (P < 0.001), and it's more likely to be diabetes patients for OA cases (P value = 0.027).

**Table 1:** Characteristics of the subjects with knee OA, and control subjects

Variables	Cases (n=500)	Controls (n=500)	P value
Age	47.8±7.8	48.2±8.2	0.430
Gender			
Male	150 (30.0%)	143 (28.6%)	0.627
female	350 (70.0%)	357 (71.4%)	
Smoking status			
Yes	78 (15.6%)	82 (16.4%)	0.730
No	422 (84.4%)	418 (83.6%)	
Alcohol status			
drinkers	178 (35.6%)	180 (36.0%)	0.895
Non-drinkers	322 (64.4%)	320 (64.0%)	
Regular physical activity			
Yes	113 (22.6%)	121 (24.2%)	0.550
No	387 (77.4%)	379 (75.8%)	
BMI	24.99±2.28	24.26±2.6	**P<0.001**
Diabetes			
Yes	114 (22.8%)	86 (17.2%)	**0.027**
No	386 (77.2%)	414 (82.8%)	

BMI=body mass index.

### Association between allelic and genotypic frequency of EN1 rs4144782 and knee OA risk

Table 2 presents the association between allelic and genotypic frequency of EN1 rs4144782 and knee OA risk. Genotype frequency of EN1 rs4144782 in the control group was in accordance with HWE (P = 0.168). EN1 rs4144782 was significantly associated with OA risk under the log-additive model (OR=1.26; 95% CI: 1.05-1.50, P value=0.012). Compared with individuals with the GG genotype, the adjusted OR for developing OA was 1.35 (95% CI: 1.03-1.77; P value=0.030) for those with the AG genotype, as well as 1.46 (95% CI: 1.03-2.06; P value=0.031) for those with the AA genotype. In dominant model, compared with carriers of GG genotype, those with AG or AA genotype have an 1.44-fold increased risk of OA (OR: 1.44; 95% CI: 1.10-1.88; P value=0.008).

**Table 2:** Associations between EN1 rs4144782 and susceptibility of knee OA among Chinese population

Genotypes	Cases (n, %)	Controls (n, %)	OR (95% CI)^a^	P value
GG	152 (30.4%)	188 (37.6%)	1.00 (Reference)	
AG	245 (49.0%)	225 (45.0%)	1.35 (1.03-1.77)	**0.030**
AA	103 (20.6%)	87 (17.4%)	1.46 (1.03-2.06)	**0.031**
A vs G			1.26 (1.05-1.50)	**0.012**
Dominant model				
GG	152 (30.4%)	188 (37.6%)	1.00 (Reference)	
AG+AA	348 (69.6%)	312 (62.4%)	1.44 (1.10-1.88)	**0.008**
Recessive model				
GG+AG	397 (79.4%)	413 (82.6%)	1.00 (Reference)	
AA	103 (20.6%)	87 (17.4%)	1.25 (0.91-1.73)	0.168

^a^ Adjusted for age, gender, smoking and drinking status, physical activity, diabetes, and BMI.

BMI=body mass index; OR=odds ratio; CI: confidence level.

### Stratified analyses of associations between EN1 rs4144782 and knee OA risk

Table 3 presents stratified analyses of association between EN1 rs4144782 and knee OA risk by age, gender, smoking and drinking status, physical activity, diabetes, and BMI. Generally, the results were not changed materially. The significant ORs ranged from 1.23 to 1.28 in allelic model (P value: 0.013-0.049). While in dominant model, the significant ORs ranged from 1.34 to 1.53 (P value: 0.007-0.049). We also detected an significant association for non-smokers in recessive model (OR:1.43; 95% CI: 1.00-2.05; P value = 0.048).

**Table 3:** Stratified associations between EN1 rs4144782 and susceptibility of OA among Chinese population

	Allelic model		Dominant model		Recessive model	
	OR (95% CI) ^a^	P value	OR (95% CI) ^a^	P value	OR (95% CI) ^a^	P value
Age group						
≥40	1.28 (1.05-1.57)	**0.013**	1.51 (1.12-2.03)	**0.007**	1.25 (0.88-1.79)	0.212
<40	1.18 (0.76-1.81)	0.461	1.24 (0.66-2.33)	0.510	1.25 (0.559-2.81)	0.583
Gender						
Male	1.34 (0.96-1.87)	0.082	1.52 (0.92-2.53)	0.103	1.43 (0.80-2.58)	0.230
female	1.23 (1.00-1.51)	**0.047**	1.40 (1.02-1.92)	**0.037**	1.19 (0.81-1.75)	0.388
BMI						
≥25	1.31 (0.83-2.08)	0.245	1.64 (0.83-3.21)	0.152	1.18 (0.51-2.74)	0.696
<25	1.24 (1.00-1.54)	**0.049**	1.38 (1.00-1.91)	**0.049**	1.27 (0.86-1.87)	0.234
Diabetes						
Yes	1.07 (0.70-1.62)	0.765	1.09 (0.58-2.03)	0.789	1.09 (0.50-2.39)	0.825
No	1.28 (1.05-1.56)	**0.014**	1.50 (1.11-2.02)	**0.008**	1.26 (0.88-1.80)	0.206
Smoking status						
Yes	1.20 (0.76-1.88)	0.436	2.13 (1.04-4.37)	**0.038**	0.66 (0.29-1.47)	0.309
No	1.27 (1.05-1.55)	**0.016**	1.34 (1.00-1.79)	**0.047**	1.43 (1.00-2.05)	**0.048**
Alcohol status						
drinkers	1.25 (0.93-1.69)	0.140	1.26 (0.81-1.96)	0.302	1.51 (0.86-2.63)	0.150
Non-drinkers	1.25 (1.00-1.55)	**0.045**	1.53 (1.09-2.15)	**0.014**	1.12 (0.75-1.67)	0.583
Physical activity						
Yes	1.11 (0.75-1.62)	0.606	1.16 (0.65-2.07)	0.623	1.12 (0.57-2.22)	0.734
No	1.28 (1.05-1.57)	**0.016**	1.49 (1.10-2.01)	**0.011**	1.28 (0.88-1.85)	0.192

^a^ Adjusted for age, gender, smoking and drinking status, physical activity, diabetes, and BMI.

BMI=body mass index; OR=odds ratio; CI: confidence level.

## DISCUSSION

In this case-control study, we investigated the association between EN1 rs4144782 and susceptibility to OA in a Chinese population. Our results showed that EN1 rs4144782 was significantly associated with increased risk of knee OA. The results reconfirmed the close connection of BMD and OA. To the best of our knowledge, this should be the first association study of EN1 locus on risk of OA.

The emerging findings of susceptibility loci of many disease have provided insight into the early diagnosis in the past years [44, 45]. OA is considered as a multifactorial disease, resulting from the combined influence of biomechanical factors and genetic factors [46]. COL2A1 was the first gene identified to be associated with OA by genetic linkage study in 1990 [47]. Following, SNPs in gene VDR, ER, TGF-beta1, GDF5, and VNTR were found in sequence [48–51]. Recently, Gok et al [52] first reported the CA repeat length of >/= 20 in the promoter region of ADAMTS9 gene has a six-fold increase in probability for having severe OA. The most intriguing hypothesis that high systemic BMD increased OA risk conclude that genetic variations associated with high BMD will lead to a corresponding increase in OA risk, which was confirmed by the detection of SNP rs2016266, rs10226308, rs3736228, and rs10835187 [43].

The engrailed gene plays an important role in the development of segmentation course, where it is required for the formation of posterior compartments [53–55]. En-1 is in the central portion of the mouse chromosome 1, while En-2 is in the proximal portion of mouse chromosome 5 [56]. Zhang et al [57] also indicate that EN1 is a positive regulator of brown adipogenesis. It was shown that polymorphisms (rs1861972, rs1861973) of homeobox transcription factor gene ENGRAILED 2 and its haplotype A-C was associated with autistic disorder in Chinese children [58]. In current study, Our results showed that EN1 rs4144782 was significantly associated with increased risk of OA. EN1 is a homeobox gene that contributes to the development in the dorsal midbrain and anterior hindbrain of human beings [59].

SNP rs4144782 was located in the intron region of EN1 gene. Using HaploReg v4 software, we found that a G to A transform will result in the expression decrement of motifs CAC-binding-protein, EWSR1-FLI1, PU.1_disc3, SP1_disc3, SP1, STAT_disc7, TATA_disc7, and TFII-I, as well as increased expression of motifs HDAC2_disc6, TCF12_disc5, and VDR_2 [60]. RegulomeDB also shows that allele exchange of EN1 rs4144782 will affect the binding efficiency of many transcription factors [61]. These will then affect the expression of En1 gene in sequence.

There are several limitations that can be derived from the present study. Although the sample size of the current study was moderate, the number of subjects on the interaction analyses was relatively small. In addition, all the patients and controls involved in this study were enrolled from the hospital, thus inherent bias may affect the results. Also, this study just considered common variation (minor allele frequency > 0.05) like other candidate gene studies. Rare variants, which owns larger effect sizes than the average effect size of common variants, should be explored for its contribution to the development of knee OA. Considering the different genetic architecture (different minor allele) of EN1 rs4144782 in different ethnicities (Asian, European and American), results should be interpreted discreetly and further replicated in other independent population with different ethnicities. Larger-scale studies examining gene-gene and gene-environment interactions are also needed.

Conclusively, in this case-control study, we identified that EN1 rs4144782 was significantly associated with increased risk of knee OA. This conclusion contributes to the hypothesis for an association of high BMD-associated SNPs with higher knee OA risk, which will help to elucidate the connection of BMD and OA, and to perform risk prediction, early prevention, diagnosis and individualized treatment of OA. Further work is needed to determine the precise role of EN1 locus on the occurrence of OA, and how rs4144782 regulates the function of EN1 gene and regulates its downstream genes, related pathways *in vitro* and *in vivo*.

## MATERIALS AND METHODS

### Study population

The case-control study was conducted in accordance with STROBE (strengthening the reporting of observational studies in epidemiology) guidelines (STROBE checklist in Supplementary File 1). The sample size was calculated using Quanto software [62]. Finally, OA cases (n=500) were recruited at Renmin Hospital of Wuhan University (Wuhan, Hubei province, China) between January 2008 and April 2015 in current study. The diagnosis of knee OA was based on having a K/L grade of 2 or higher in at least one knee [63]. The representative X-ray images were presented in Supplementary File 2. Healthy controls (n=500) were randomly selected from healthy persons under routine health Screening during the same study period. Propensity-score matching was used to select controls to minimize selection bias due to perceived confounders, with the following variables as contributors to the propensity score: age, gender, smoking status, alcohol status, and physical activity. To eliminate the potential recall bias, the participants were interviewed face to face using standard questionnaire. A 5% samples were checked for the concordance. Ethical approval was granted by the Renmin Hospital of Wuhan University research ethics committee, and was conducted in compliance with the Declaration of Helsinki. Informed written consent for blood use and data publication was provided by all donors.

### DNA extraction and genotyping

Peripheral venous blood (2ml) from all participants was collected by vacuum tube with ethylene diamine tetraacetic acid (EDTA). Genomic DNA was extracted from the whole blood samples of all participants using QIAamp DNA Blood Mini kit (Qiagen, Berlin, Germany) according to the manufacturer's instructions. The DNA concentration and purity were estimated by measuring ratio of the absorbance at 260 and 280 nm. The tagSNP rs4144782 was selected using the web-based software SNP info (https://snpinfo.niehs.nih.gov/). Genotyping was done with Sequenom iPLEX matrix assisted laser desorption/ionization–time-of-flight mass spectrometry technology using 15 ng DNA. The MassARRAY Typer 4.0 software was used for proper data acquisition and analysis. A random 10% of quality control samples were checked for the concordance. Laboratory personnel were blinded to the case-control status of the specimens, and to the quality control samples.

### Statistical analyses

All analyses were performed using SAS version 9.2 and P values were two-sided in current study. The Hardy–Weinberg equilibrium (HWE) was assessed using a goodness-of-fit χ2 test performed to identify possible genotyping errors among the controls of each study. Categorical variables were compared between patients with knee OA and healthy controls using the χ2 test or Fisher's exact test where appropriate. Logistic regression was used to estimate ORs and 95% confidence intervals (CIs) as a measure of the association with the susceptibility of knee OA, adjusted for the potential confounding factors, including age, gender, smoking and drinking status, physical activity, diabetes, and BMI. We also conducted subgroup analyses by age, gender, smoking and drinking status, physical activity, diabetes, and BMI.

## SUPPLEMENTARY MATERIALS FIGURES AND TABLES




