# Supplementary material for: Association between EN1 rs4144782 and susceptibility of knee osteoarthritis: A case-control study
Source: Oncotarget. 2017 Apr 5;8(22):36650–7. doi: 10.18632/oncotarget.16842 (PMC5482684; doi:10.18632/oncotarget.16842)
Supplement: Supplementary file 1 [file oncotarget-08-36650-s001.pdf]

Association between EN1 rs4144782 and susceptibility of knee osteoarthritis: A case-control study

SUPPLEMENTARY INFORMATION

Supplementary File 1: STROBE Statement. Checklist of items that should be included in reports of observational studies

See Supplementary File 1

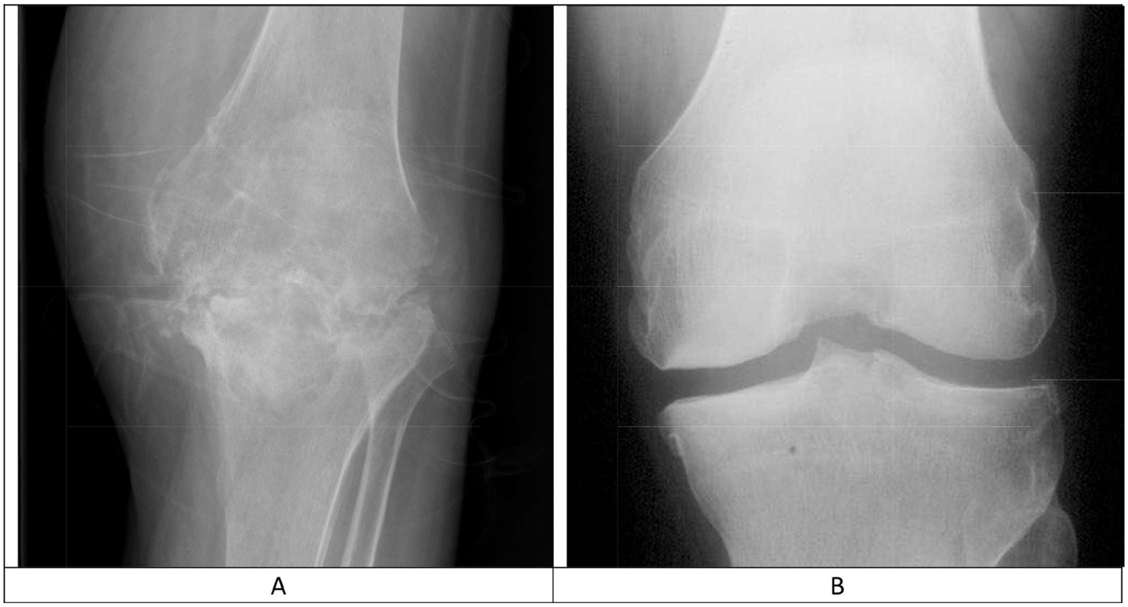

Supplementary File 2: X – ray of OA case and control. **(A)** OA case; **(B)** Control.
